# A critique of the regulation of data science in healthcare research in the European Union

**DOI:** 10.1186/s12910-017-0184-y

**Published:** 2017-04-08

**Authors:** John M. M. Rumbold, Barbara K. Pierscionek

**Affiliations:** 1 Faculty of Science, Engineering and Computing, Kingston University London, Penrhyn Road, Kingston upon Thames, KT1 2EE UK; 2 School of Science and Technology School of Science and Technology, Nottingham Trent University, 50 Shakespeare Street, Nottingham, NG1 4FQ UK

**Keywords:** Big Data, Data science, Research Ethics, Information governance

## Abstract

The EU offers a suitable milieu for the comparison and harmonisation of healthcare across different languages, cultures, and jurisdictions (albeit with a supranational legal framework), which could provide improvements in healthcare standards across the bloc. There are specific ethico-legal issues with the use of data in healthcare research that mandate a different approach from other forms of research. The use of healthcare data over a long period of time is similar to the use of tissue in biobanks. There is a low risk to subjects but it is impossible to gain specific informed consent given the future possibilities for research. Large amounts of data on a subject present a finite risk of re-identification. Consequently, there is a balancing act between this risk and retaining sufficient utility of the data. Anonymising methods need to take into account the circumstances of data sharing to enable an appropriate balance in all cases. There are ethical and policy advantages to exceeding the legal requirements and thereby securing the social licence for research. This process would require the examination and comparison of data protection laws across the trading bloc to produce an ethico-legal framework compatible with the requirements of all member states. Seven EU jurisdictions are given consideration in this critique.

## Background

The improvement of healthcare can be accelerated by an information governance framework that facilitates audit and research across an entire region [1]. The European Union (EU) offers an opportunity to maximise the benefits of harmonisation of healthcare across different languages, cultures, and jurisdictions (albeit with a supranational legal framework), enabling improvement of healthcare by comparisons across the entire supranational trading bloc. This process would be facilitated by the examination and comparison of data protection laws and ethical requirements across the EU to produce an ethico-legal framework compatible with the requirements of all member states. This process is a fundamental part of achieving a Digital Single Market and integral to the free movement of services [2]. Patients are becoming more mobile with health tourism a major issue for regulators. Facilitating comparisons between providers in countries will enable genuinely informed choices to be made by consumers.

Big Data is a term that has been used broadly in the academic literature and has been characterised by the 5 Vs: volume, velocity, variety, veracity and value [3]. It involves the integration of large volumes of data from various sources that are gathered speedily to inform timely decision-making. The data need to be reliable and provide some benefit. Big Data has enormous potential for performing large-scale epidemiological studies at low cost and improving the quality of healthcare via the large quantity of healthcare data gathered routinely [4, 5]. However, the term Big Data is often misused to simply mean the analysis of large datasets. The issues of scale alone do not warrant any special treatment; Big Data is often used to simply mean data science.

## Main text

This critique applies to the use of all forms of data for healthcare research, particularly the secondary use of healthcare data because the use of Personally Identifiable Information (PII) without consent is both an ethical and legal issue. Where Big Data is being used directly for healthcare improvement, this will normally come under the rubric of the ordinary use of medical records for management of healthcare services, and therefore does not require consent. However, this justification in the case of the Google DeepMind project being run with the Royal Free NHS (National Health Service)Trust has been questioned, and the Information Commissioner’s Office obliged to investigate after complaints were made [6]. The dividing line between audit and research is blurred [7] and the advent of data science arguably makes this blurred line even more significant since Big Data makes research on routinely collected data much more practicable, as opposed to the traditional reliance on datasets collected specifically for research purposes. If the activity is deemed to fall under the research category and the data is still PII, consent must be sought unless an applicable exemption can be applied. The laws and ethics of healthcare data include provisions where the gaining of specific informed consent will be impracticable and the research is in the public good. The relevant laws leave the process of determining which research will be exempt implicitly down to Research Ethics Committees (RECs) or the equivalent, often in combination with data protection agencies. In the UK, the approval of both RECs and the Confidentiality Advisory Group are required for the use of NHS data. In Italy, the *Garante* is required to authorise healthcare data use even when consent has been obtained, unless the research comes under the general waiver (Article 110 of the Personal Data Protection Code).

Alternatively, data can be anonymised. Anonymisation renders data no longer personally identifiable in law. The legal standards do not require re-identification to be impossible as any such requirement would render the processed data almost useless [8]. Anonymisation ought therefore not to be seen as an all-or-nothing process, but rather a procedure to reduce the risk of re-identification to an acceptable level. The risk of re-identification is dependent on a number of factors, which makes the imposition of a single legal standard problematic. For example, it is problematic trying to determine whether a dynamic Internet Provider Address (IPA) is PII or not, as the case of *Breyer v Germany* demonstrates [9]. The anonymisation framework published by the UK Anonymisation Network (UKAN) emphasizes the difficulty in formulating a comprehensive guideline [10]. Anonymisation prevents the linking of records and elimination of duplicate records. Where records are identified by a unique code, this can be prevented and is termed pseudonymisation.

RECs and their equivalents often lack expertise in data protection law and related issues such as the difficulty in defining anonymisation. Much medical research is performed on data gathered specifically for research purposes and Big Data offers further opportunity to perform an enormous variety of research on data already gathered. Data protection laws offer protection for users regarding secondary uses, with consent being the usual mechanism. However, there are derogations for research of public benefit. This is especially important for Big Data projects, where gaining individual and specific consent poses significant logistical difficulties.

Given the wide-ranging protection for the autonomy, privacy and dignity of data subjects by the relevant laws, RECs arguably could and should have a light touch when faced with applications that deal with such issues. The main factor where the authors consider that RECs ought to require more protection than necessarily required by data protection law is anonymization as in certain countries, notably in the UK, the legal standard is not suitable for the protection of the subjects of medical research.

The current data protection regime in the EU is based on the Data Protection Directive. National transpositions of the Data Protection Directive have varying standards for anonymisation (Table 1). Where the relevant ethico-legal standards are unclear, there is the potential for unnecessary regulatory delay for projects. The General Data Protection Regulation (GDPR) will apply from June 2018; regulations are directly applicable, so that citizens can rely on them for protection of rights. The GDPR will not apply if and when the UK exist the European Union, although planned legislation would enable its continued enforceability (the so-called “Great Repeal Act”) [11].

**Table 1:** Variations in Data Protection for Medical Research between the seven EU Countries

	France	Germany	Greece	Italy	Nether-lands	Sweden	UK
Is informed valid consent sufficient?	YES	YES	YES	NO - also requires approval by the Garante	NO – professional duties of confidentiality may override consent	YES	YES
Is broad consent permissible?	YES	YES	YES	NO	NO	NO	YES
Definition of anonymization	All means available to controller or other person must be considered (CNIL approve means of anonymisation)	Identification not possible without disproportionate time and effort	No definition in statute, but supervisory authority applies Recital 26 definition	Identification not reasonably likely, no identification numbers	Identification reasonably excluded	Cannot be identified by someone even with considerable time, effort or other resources	Defined by ICO – currently the ‘motivated intruder’ test
Is pseudonymised data^a^ treated as anonymised?	CNIL guidance suggests if key code kept secret, YES	Only for third parties without the key code	Probably YES	Probably for third parties without the key code	YES	NO	Only for third parties without the key code

^a^Reversible pseudonymisation by key code, rather than irreversible eg by one-way cryptography

### EU data protection law

EU data protection acts all have the same basic features but with some national differences as illustrated in Table 1. This permits the adjustment of protections according to the attitude of publics. For example, Denmark has used a system of broad consent to promote legitimate research using genetic data on the basis of an “opt-out” system which assumes that an individual consents unless he or she says otherwise [12]. Recently, it was revealed that a large amount of healthcare data had been retained illegally in the Danish General Practice Database (DAMD), as the limited ambit of the original scheme had been exceeded [13].

The protections for privacy will vary from member state to member state, according to both the implementation of the Directive and the ‘reasonable expectations’ of the public. The applicable regulations and supervisory body are of the country where the data controller has its establishment. The nationality of the subject is immaterial (as per *Weltimmo v Nemzeti Adatvédelmi és Információszabadság Hatóság*). The GDPR will bring in a “one-stop shop” regime for national data protection authorities (Recital 127). This means that data projects cannot be facilitated by simply transferring data to the EU member state with the weakest protections, even if this were deemed ethical.

Although member states are not able to increase the level of protection, they can widen its scope. The Opinions and reports of the Article 29 Working Party have helped to develop a shared understanding of the interpretation of the terms in the Directive. The UK approximation is the Data Protection Act 1998. Article 7 lays out the conditions for lawful processing of personal data:

Member States shall provide that personal data may be processed only if:

- the data subject has unambiguously given his consent; or
- processing is necessary for the performance of a contract to which the data subject is party or in order to take steps at the request of the data subject prior to entering into a contract; or
- processing is necessary for compliance with a legal obligation to which the controller is subject; or
- processing is necessary in order to protect the vital interests of the data subject; or
- processing is necessary for the performance of a task carried out in the public interest or in the exercise of official authority vested in the controller or in a third party to whom the data are disclosed ; or
- processing is necessary for the purposes of the legitimate interests pursued by the controller or by the third party or parties to whom the data are disclosed, except where such interests are overridden by the interests for fundamental rights and freedoms of the data subject which require protection under Article 1 d).

These restrictions apply to personal data only and there are limited derogations for the use of personal data for research purposes without consent. Exemption only applies where consent is not “reasonably practicable” and the research is “in the public interest”. Healthcare management and audit is a compatible purpose, and therefore does not require consent. Table 1 compares seven member states on the issues of anonymisation and consent:

### The General Data Protection Regulation (GDPR)

The GDPR is enforceable from mid-2018.[14] The important clauses in the GDPR are:

1. Specific provision for categorical consent to research
2. Specific categorization of research as a compatible purpose
3. Derogation from prohibition on processing of sensitive data for research
4. Clarification that pseudonymised data is still personal data.
5. Broadening of the definition of personal data

Exactly how broadly “certain areas of research” (Recital 33) should be interpreted is not clear. “Recognized ethical standards” certainly allow for subjects to consent to projects with a similar risk. When there is a significantly different risk (quantitatively or qualitatively), it may extend outside the remit of the broad consent. This is an area where continuing governance with subject and/or public input can usefully guide decision-making.

The derogation for scientific research on healthcare (and other sensitive) data applies only to research in the public interest (Recital 52). It is not clear whether or not this might exclude all commercial projects, or only those where there are no benefit sharing arrangements. Presumably this is a different test from Section 9.2 g where “substantial public interest” is required. The Confidentiality Advisory Group (CAG) in the UK has publicised its interpretation of “public interest”, which requires a balance between harms and risk, with the aim of determining what is likely to be reasonably acceptable to the public [15].

The definition of personal data has broadened. Although the types of data that might lead to the individual being identifiable are stipulated, personal data themselves are any data that relate to such an identifiable individual. This is part of a global trend [16–18]. This reaction to the possibility of re-identification has the potential for decreasing incentives to use techniques such as anonymisation and pseudo-anonymisation.

There is allowance for differences and a margin of manoeuvre for member states to specify rules, including those that pertain to processing sensitive data, the circumstances of specific processing and setting out conditions that determine whether or not processing of personal data is lawful (Recital 10).

### Data use in research

There is a fundamental need in long-running projects for an effective governance mechanism that has suitable and effective input from research subjects. The one-off granting of ethical approval is insufficient to guarantee research subjects the protection that they should have. Given the difficulties predicted by Collingridge about the regulation of technology, [19] a reflexive governance framework backed by law would be preferable to cumbersome legislation given its greater ability to adapt to emerging challenges and technologies. The need to make more use of the law for promotion of public interest has been highlighted [20].

The law often takes an individualistic approach which can fail to take sufficient account of the wider public benefits of Big Data and data science. A governance framework for data science that better reflects the altruistic intentions behind participation is needed. This would enable participation, whilst providing appropriate protection from exploitation. The GDPR promises to better harmonise data protection law in the EU as it is directly applicable, but there remains flexibility for member states to vary protection. Although differences in data protection law of member states should not hinder data transfer (Article 1.2 of the Data Protection Directive), this legal doctrine may not reassure RECs that are faced with the potential of data transfer to member states with lower protections than those found in their own. Additionally, the regulations do not address in detail the ethical issues. Although it is entirely appropriate that regulations are not too prescriptive on an evolving area of ethics and law, there are concerns that decisions made by RECs are either too variable or too restrictive to prevent this flexibility from being exploited. A useful framework for considering data research is one based by three pillars – anonymisation, consent, and governance. If one pillar is weak, the other two have to be stronger.

There is a large body of academic literature on biobanking that is highly relevant to the ethico-legal framework for Big Data in healthcare. A notable similarity is the difficulty in gaining consent when future projects cannot be predicted but the risks to individuals remain low. A clear difference is that individuals have usually been enrolled into a biobank and given consent for their information to be used for research, although as a panel discussion at the 2016 UK Biobank 1st Annual Meeting revealed, the exact ambit of to what exactly participants had consented was not clear to all. There was some debate over whether or not biopsy samples were part of the medical record, and therefore whether further consent was necessary to do research on these clinical samples.^1^ An analogous situation is the reuse of heelprick blood samples (taken routinely from newborn babies to screen for certain diseases such as phenylketonuria) for further research [21, 22]. Another important difference between biological samples and standard medical record data is that genetic data derived from these samples are seen more as a community resource than personal healthcare data [23–26]. However, both are considered sensitive personal data requiring special protection and permission for their usage.

### Regulatory barriers

The restrictions on the use of health data for research can be categorized thus:

**Legal**: Statutory and case law restrictions on use of data or invasion of privacy will involve remedies and/or punishments for infringement. There is a common law duty of confidentiality in the UK and the right to privacy under Article 8 of the European Convention on Human Rights (ECHR). The UK law has to be interpreted in line with the EU Data Protection Directive (where directly effective), the EU Charter of Fundamental Rights, and the ECHR. The GDPR will be directly applicable.

**Ethical/moral**: The application of healthcare ethics needs to consider the major principles of beneficence, non-maleficence, respect for autonomy and confidentiality. In the UK, a general practitioner is required under the Health and Social Care Act 2012 to disclose patient details; General Medical Council requirements and common law confidentiality duties permit these disclosures to fulfil statutory duties GPs have been threatened with termination of their contract over plans to opt all of their patients out of Care.data) [27].

Moral considerations concern subjective personal beliefs about right and wrong. It has been argued that there is a moral duty to engage in research [28]. The UK Medical Research Council report found 60% of the public felt a responsibility as beneficiaries of medical research to participate in medical research provided suitable safeguards were in place [29]. It might be considered immoral to exploit patients by selling their healthcare data to commercial concerns (as proposed in the Care.data project).

**Professional**: Professional regulators and other professional craft bodies may impose stricter duties of patient confidentiality than the law requires – but there is a duty to comply with statutory requirements.

**Cultural**: Data sharing that is legally, ethically and professionally acceptable may nonetheless be considered unacceptable by service users [30]. Although they may have no formal means of redress, their objections may lead to widespread opting out (if available) or a reduction in trust between healthcare professionals and patients. Withholding information from healthcare professionals reduces the veracity of health records and risks detrimental effects to health. UK consultations suggest a considerable proportion of the population support the use of their data to improve healthcare whether via audit or research [29, 31–33].

### Ethico-legal issues with data science

The ethical issues that need to be addressed with data science generally are:

1. “Ownership” of the data
2. Autonomy of the individual
3. Privacy/confidentiality
4. Necessity and proportionality; beneficence/non-maleficence

If these ethico-legal concerns are not addressed properly, then any projects will face problems with public trust.

### “Ownership” of the data

There is no property in data (*Oxford v Moss, Your Response Ltd vs Datateam Business Media Ltd*). Yet a database can be intellectual property and therefore intangible property in English law, and can therefore be protected by copyright. The 2013 Caldicott review of information governance in the UK confirmed that patients do not own their NHS data [34–36] (although there can be patient-owned records, and the ownership of data generated by medical devices for the ‘Internet of Things’ (IoT) is an interesting issue that requires further exploration [37]).

The situation in non-common law countries (where law cannot be made by a court without the necessary statutes) appears to be the same – there are intellectual property rights in a database (which may be copyright or the database right mentioned above), but no property in data *per se*. The Database Directive 96/9/EC provides a *sui generis* right for database owners to protect databases not seen as intellectual property due to the lack of originality.

The passage of the UK Health and Social Care Act 2012 placed the disclosure of certain data to NHS England outside the main provisions of the Data Protection Act, which was the UK implementation of the Data Protection Directive, and also exempted GPs from the common law duty of confidentiality when disclosing data to the Health and Social Care Information Centre (HSCIC). The framework for Care.data arguably contravened the provisions of the GDPR. The Care.data project was stopped but the framework remains in place for the secondary use of patient data for research.

Some commentators have advocated that the patient should (and can) have ownership of their healthcare data.[38] They argue that the patient holding a personal health record would counter the obstacles to sharing information in the interest of the patient and safe health care. Other commentators believe that the notion that subjects have even partial interests in their health data or in bio-specimens would hinder research [35, 36, 39, 40]. Given that altruism is a major motivator of involvement in medical research, this is not generally problematic – except when a small community is being studied e.g. those with a rare genetic disorder. Here it is notable that patients and families have in some cases created disease-specific biobanks, where they retain legal control over the materials [24]. Where the outputs are expected to be of benefit to the wider public, involvement of representatives of the general public must be considered [20]. Altruism as a motivation feeds into public expectations about uses for healthcare data.

### Autonomy

Returning to the similarities with biobanks, Laurie comments that the consent standard for clinical trials, informed consent, is not possible with a long term project where the ambit of future individual research studies cannot be predicted [41]. Further, the ScottisH Informatics Programme (SHIP) found that an informed public accepted broad consent as suitable and practical [31]. A US study confirmed that most subjects (66%) would prefer a wider form of consent to study-specific consent. The options considered were consent to any future research projects authorised under an appropriate governance mechanism, and categorical consent [42]. Categorical consent is a type of broad consent but limited to certain nominated categories of study. There is also the option of dynamic consent [43]. All forms of broad consent need to have robust mechanisms for withdrawal of consent (except dynamic consent). This need not be retroactive in effect [20].

Regulation of medical research has the purpose of protecting subjects who by definition are vulnerable. This entails a degree of paternalism by definition, otherwise it would be sufficient to simply rely on informed consent. Some have argued that the paternalism demonstrated by RECs inappropriately denies research subjects the opportunity to take risks they find acceptable. Edwards et al. make the point that research subjects who are considered autonomous should be entitled to decide on which research they participate in rather than have their choices constrained by a REC; this is considered to be overprotective and paternalistic [44]. An exception would be the prohibition on financial inducements with regard to recruitment. Garrard and Dawson disagree with this on the grounds of consistency with regards to antipaternalism [45]. The nature of these projects entails some level of risk for the data to be useful; rigorously anonymised data that excludes the possibility of re-identification at a later date is almost useless. This is where the concept of differential privacy is useful, as it demonstrates the background risk to privacy from information already in the public domain. The risks of damage distress relate to data breaches or misuse of information [30].

The nature of Big Data projects may make gaining informed consent impractical. If consent is not obtained, opting out must be a practical possibility – which requires publicity and education of the public so that such a right can be exercised. The use of anonymised data requires no consent, but certain types of research require records to have an identifier. The main mechanism for accommodating both requirements is pseudonymisation, where typically identifiers are replaced with a unique identifier. The patient can only be re-identified with the aid of a key code. Such pseudonymised data may or may not be considered as personal data depending on the circumstances and the member state – in particular, third parties without access to the key code may or may not be able to treat the data as anonymised depending on the test applied (Table 1). This is clarified in the GDPR, which classifies all pseudonymised data as personal (Recital 26).

Consultations for SHIP have shown that personal control over health data is considered important even when data is anonymised [31]. This issue is relevant to social license rather than legality *per se*. Some people might find the use of their anonymised data objectionable and this could occur if the particular research project offended their morals. In such cases it is difficult to see what right is at stake, although some have argued that even the sharing of anonymised healthcare data is breaching confidentiality [46].

### Privacy/Confidentiality

The use of information technology in hospitals facilitates delving into personal data on a scale not possible with physical hospital records. There have been well-publicised breaches of confidence of celebrities in the USA; in one case, a notable American footballer’s medical records were viewed by 1,754 separate employees during an admission [47].

There have been several large-scale hacks (over 1 million individuals affected) of medical records in the USA [38]; one affected 80 million individuals. A recent study found major data security flaws in many accredited health apps available via the UK NHS Choices Health Apps library [48]. There have notable disastrous consequences from central government handling of sensitive data, and the NHS has a relatively poor record on data protection – it was deemed to be the worst organization in the UK for serious data breaches in 2014 according to ICO statistics [49]. USB sticks have been lost, [50] hard drives with patient data on sold on EBay, [51] and faxes and emails misdirected [52, 53]. Whether or not these lapses are reflected in public attitudes is questionable – an IPSOS Mori poll found that the NHS fared relatively well in terms of public trust. Trust on data protection in the NHS was 36%, and in GPs 41% but media, internet companies, telecommunications companies and insurance companies were trusted by only 4–7%.[54] The Centre for Media, Data and Society looked at data breaches across Europe and found that Germany, Greece, the Netherlands, Norway and the UK had the greatest number of reported breaches [55]. Overall 2% of these breaches involved medical organizations.

The two main protections for subject privacy are data security and anonymisation. Data security is an integral part of the ethico-legal framework, and appropriate technology, procedures and training are essential to ensure that good data security standards are maintained. Anonymisation removes the consideration of data as personal and hence any coverage of it by data protection legislation (many of the member states’ statutes are *personal* data protection acts).

The definition of personal data in Recital 26 of the Directive states:

to determine whether a person is identifiable account should be taken of all the means likely reasonably to be used either by the controller or by any other person to identify the said person [56].

It adds that:

data may be rendered anonymous and retained in a form in which identification of the data subject is no longer possible

This has been interpreted to mean that anonymisation must be irreversible, which rules out pseudonymised data as being anonymised under the Directive. One-way cryptography is treated as anonymous (subject to certain caveats), because it is irreversible. The test for anonymisation in the UK refers to information held by the data controller, therefore data that is provided to researchers who do not have the key code can be treated as anonymised.

*R v Department of Health, ex parte Source Informatics* establishes that the provision of anonymised information does not breach the common law duty of confidentiality. *Common Service Agency v Scottish Information Commissioner* confirms that anonymised data does not come under the provisions of the Data Protection Act; however, the case avoids the issue of what constitutes anonymisation. The data in this case was requested to be released after ‘Barnardisation’,^2^ but this weak form of disguise would not necessarily prevent re-identification given the nature of the data sets. The House of Lords remitted the matter of whether or not the data were anonymised or not back to the Scottish Information Commissioner to decide, as an issue of fact not law. This deference on the issue means that the pronouncement by the UK Information Commissioner’s Office (ICO), that anonymisation is deemed effective when the risk of re-identification is remote rather than absent, nevertheless recognises the small risk of re-identification.

The test applied does not vary according to the context or the sensitivity of the data. Re-identification is a challenge that older regulations fail to address adequately, although the Data Protection Act provides that personal data is:

data which relate to a living individual^3^ who can be identified—

(a) from those data,

or

(b) from those data *and other information which is in the possession of, or is likely to come into the possession of, the data controller*, and includes any expression of opinion about the individual and any indication of the intentions of the data controller or any other person in respect of the individual [italics authors’].

The Confidentiality and Security Advisory Group for Scotland stated that:

100% anonymity is almost impossible to achieve without the data set being reduced to one data item, rendering it of little use for most research purposes [8].

There are a number of different standards of anonymisation applied across Europe. The ICO states that the potential for re-identification is:

essentially unpredictable because it can never be predicted with certainty what data is already available or what data may be released in the future [57].

The same uncertainties are behind the Australian Privacy Commissioner’s statement about treating anonymised data as personal information [16]. However, the ICO’s assessment did not affect the decision in FS50565190 against Queen Mary University of London relating to the PACES trial [58]. The ICO held that the University could not withhold data that had been anonymised to the ICO’s satisfaction despite concerns about activists trying to re-identify participants. The ICO wanted specific explanation as to how re-identification would be achieved. It can be persuasively argued that the motives of those wishing to use the data should affect the level of anonymisation required.

A further solution to the problem of re-identification is to restrict access to researchers who have given assurances they will not attempt re-identification. These currently make no difference to whether or not the data is classified as indirectly identifiable or not, despite the benefits of such arrangements. They are an integral part of information governance arrangements for data “safe havens” [59]. In the UK, Accredited Safe Havens are “contractually and legally bound to process data in ways that prevent the identity of individuals to whom the data relates from being identified” [60]. There are similar mechanisms in Ontario, Canada for “prescribed entities” where approved policies must be in place to protect data (the Institute for Clinical Evaluative Sciences specifically states this in their privacy policy) [61]. Additionally, any data that has been re-identified becomes PII as a matter of law. The likelihood of re-identification is contested, [62] but the conclusion of both the Nuffield Council on Bioethics and SHIP is that the ‘consent or anonymise’ approach is not sufficient to guarantee the protection of subjects’ interests [63, 64]. Data security can be more important than anonymity. Existing data available about a subject pose a background risk to privacy, and differential privacy is a technique for assessing whether the research will increase that risk or not. The reasonable expectations of the public are that a minimal risk is acceptable, particularly if measures are in place to reduce risks.

### Necessity and proportionality

All the rights protected by the European Convention on Human Rights, bar Article 3 (prohibition of torture, inhumane or degrading treatment or punishment), are qualified rights, so interference with Convention Rights is permitted when certain criteria are met. The United Kingdom is obliged as a contracting party to ensure the compatibility of British law with the upholding of Convention Rights. In *Handyside v United Kingdom,* the European Court of Human Rights held that this needed in order to be necessary and proportionate. “Necessary” implied a ‘pressing social need’. Exemptions have to be proportionate to the legitimate aim pursued, and the reasons given have to be relevant and sufficient.

Several documents have sought to emphasize the public interest in benefitting from audit and research as a justification for interference with the rights to privacy and autonomy. This needs to be assessed on a case-by-case basis, which entails the adoption of the principled proportionate governance model or something very similar. The Directive requires a ‘substantial public interest’ for member states’ exemptions above and beyond the Directive’s requirements, and it seems perilous to rely on the public interest *per se* to justify routine epidemiological research using personal health data. Indeed, a report for the European Commission indicated that this sort of justification may be violation of the Directive [65].

In practice, the ICO and other regulatory bodies in the UK tend to default to the “consent or anonymise” model (partly due to the provision of rights to private life under Article 8 [1] of the ECHR).

The beneficence/non-maleficence considerations for research data banks are similar to those for biobanks. The main risk is of disclosure of sensitive information. It has been shown that the combination of three pieces of data could identify 87% of US residents – five-digit zip code, birth date, and gender [66]. Anonymisation to a sufficiently high standard eliminates the risks, but also reduces the usefulness of the data. Thus there is a balancing act for any project that is best achieved through a human rights approach. There are different levels of sensitivity even within the category of healthcare data [67]. Differential privacy is particularly helpful in this regard [68].

### The Principled Proportionate Governance Model (PPGM)

The SHIP engaged with the public in a far more effective way to determine what the *reasonable expectations* of their population were, in marked contrast to the ill-fated Care.data project in England. This secured the social licence for the use of personal health data and did not exclude the use of public data for commercial purposes subject to certain caveats [31]. The recent Wellcome Trust-commissioned study had similar findings [69]. The reasonable expectations of the population help determine what compatible purposes are (Article 6(b) of the Directive).

Their findings were that in line with principled proportionate governance, projects that do not fit the ‘consent or anonymise’ could be authorised under the statutory framework by the Privacy Advisory Committee (the CAG is the relevant body in England and Wales). Therefore, the paradigm would be “consent, anonymise or authorise” (addressing the three pillars mentioned above).

Getting data protection law right is vital for healthcare improvement in the EU; it will ensure the trust of citizens in the security and confidentiality of the healthcare data and their continued support for medical research. The potential for a massive acceleration in improvements in patient safety can be realised. The large (and unexpected) number of opt-outs from Care.data illustrate the penalties for getting it wrong [70, 71]. At its worst, data issues can lead to patients lacking confidence in their healthcare provider and avoiding public healthcare altogether [72]. The public need to be involved in a meaningful way, and this requires education on the issues. Transparency about the risks involved is vital, and the majority of the public accepts a small risk if there are appropriate measures in place to mitigate them. The tricky balancing act between data utility and privacy can be achieved via proportionate governance mechanisms. This and a commitment to research in the public interest will ensure that there is a social licence for Big Data healthcare research [73].

## Conclusions

There is divergence between EU jurisdictions on dealing with the ethico-legal aspects of data for healthcare research. The key issues are those that respect autonomy and privacy notably, the requirements for informed consent and anonymisation. The increasing focus on data sharing brings tensions with the demands of data protection. Participants in a clinical study might rightly feel (as may the researchers) that a higher standard of anonymisation than usual is appropriate, or that different standards should be appropriate for the use of researchers compared with public release.

Whatever framework is in place, the evidence from previous studies supports continuing public engagement within the governance mechanism to ensure that the requirements for social licence are fulfilled and the research community continues to deserve the trust of society. A one-off process of obtaining consent can no longer be considered sufficient in all circumstances, especially with long-term ongoing Big Data projects. Subjects can no longer be seen as passive and reciprocity must be maintained. Public engagement must include the wider public not just research participants where the results will be of benefit to the wider general public.

## Abbreviations

CAG: Confidentiality Advisory Group
CNIL: Commission nationale de l’informatique et des libertés
DAMD: Danish General Practice Database
ECHR: European Convention on Human Rights
EU: European Union
GDPR: General Data Protection Regulation
HSCIC: Health and Social Care Information Centre
ICO: Information Commissioner’s Office
IoT: Internet of Things
IPA: Internet Provider Address
NHS: National Health Service
PII: Personally Identifiable Information
PPGM: Principled Proportionate Governance Model
REC: Research Ethics Committees
SHIP: ScottisH Informatics Programme
UKAN: UK Anonymisation Network

## Footnotes

1 Personal observation by first author who attended.

2 A process of randomly adjusting figures in a dataset by 1,0, or −1.^37^

3 The DPA unlike many European equivalents does not cover deceased persons; however, the common law of confidentiality may apply.
